# Profile of tuberculosis patients with comorbid diabetes mellitus in Medan, Indonesia: a cross-sectional study

**DOI:** 10.11604/pamj.2024.49.54.36492

**Published:** 2024-10-24

**Authors:** Rina Amelia, Isti Ilmiati Fujiati, Dharma Lindarto, Em Yunir, Hari Kusnanto

**Affiliations:** 1 Department of Community Medicine, Faculty of Medicine, Universitas Sumatera Utara, Medan, Indonesia,; 2 Department of Internal Medicine, Faculty of Medicine, Universitas Sumatera Utara, Medan, Indonesia,; 3 Department of Internal Medicine, Faculty of Medicine, Universitas Indonesia, Jakarta, Indonesia,; 4 Department of Public Health Faculty of Medicine, Universitas Gadjah Mada, Yogyakarta, Indonesia

**Keywords:** Tuberculosis, comorbid diabetes mellitus, blood glucose levels, lipid profile, chronic complications

## To the editors of the Pan African Medical Journal

Tuberculosis (TB) is a neglected disease and poses a greater risk in vulnerable socioeconomic groups and people with other diseases, such as HIV, alcoholism, chronic lung disease, cancer, malnutrition, and diabetes mellitus (DM) [1]. Diabetes mellitus is known to be a critical comorbid in the development of TB. Patients who have DM are at 2-3 times higher risk of developing TB than those who do not have DM. Studies conducted in different parts of the world show that 12-44% of TB diseases are related to DM [2]. In Indonesia, TB became the second-highest cause of death after stroke and the number one killer among infectious diseases. As mentioned before, people with DM are at a higher risk of developing TB than those without DM. Research shows that in countries with an increasing prevalence of DM, the prevalence of TB is also increasing. Tuberculosis and DM are among the top 10 leading causes of death in lower-middle-income countries. The health burden due to TB will keep on increasing, concerning the increasing prevalence of DM [3].

The relationship between DM and TB is a fundamental challenge in global TB control; patients with both conditions can show a high rate of TB treatment failure, increasing the risk of relapsing and even death. TB with DM may develop resistance to drugs utilized [4]. Because of their immune dysfunction, people with DM are more likely to contract new tuberculosis infections and reactivate the disease, develop active disease, and experience poor treatment outcomes [5]. Diabetes mellitus directly impairs the innate and adaptive immune responses required to fight the progression from infection to clinical manifestation. The link between DM and TB is supported by DM patients' cell-mediated immune disorders, kidney failure, micronutrient deficiency, and pulmonary microangiopathy, all of which increase their susceptibility to developing TB [6,7]. Thus, with the aforementioned explanation, this study aimed to find out the prevalence of TB with comorbid DM, and it was constructed based on age and gender in Medan, Indonesia.

The research was conducted at 77 primary health care facilities in Medan and it obtained a number of 762 TB patients with DM comorbidity. The highest number of TB with DM cases was found in Advent General Hospital and Medan Johor Primary Health Care (PHC), with as many as 43 cases, followed by 35 cases in Pulmonary Specialist Hospital, 30 in Belawan PHC, 29 in Amplas PHC, and 21 in Helvetia PHC.

Table 1 shows a total of 762 patients, 523 (68.6%) were male and only 239 (31.43%) were female. The most prevalent ages fell in the ages of 45-54 years, accounting for 271 (35.6%) respondents, followed by the age group of 55-64 years for 229 (30.1%), and the age group of 35-44 years for 134 (17.6%). Meanwhile, the least ages were in the age group of 0-4 years for only one patient and no TB patient was aged 5-14 years old. From several Asian studies, the prevalence of DM with TB coinfection was between 5-50%, with the incidence rates in DM patients increasing 1.8-9.5 times higher than those in the general population [7].

**Table 1:** frequency distribution of tuberculosis with comorbid type 2 diabetes mellitus by gender and age group

Demographic Characteristics	Frequency	Percentage (%)
**Male (n=523)**		
0-4	0	0
5-14	0	0
15-24	8	1.5
25-34	21	4.0
35-44	98	18.7
45-54	188	35.9
55-64	150	28.7
≥65	58	11.1
**Women (n=239)**		
0-4	1	0.4
5-14	0	0
15-24	2	0.8
25-34	10	4.2
35-44	36	15.1
45-54	83	34.7
55-64	79	33.1
≥65	28	11.7

The majority of TB patients with comorbid DM were male (68.6%). This result is in line with Alebel *et al*. in India, who reported higher TB incidence with DM in male patients; of 300 patients, 183 (61%) were men, and 117 (39%) were women [7]. However, other studies stated that DM patients were dominated by women (58.3% in Asia and 54.1% in Africa) [7], while another showed no significant differences between men and women [8].

Diabetes may also increase the number of *Mycobacterium tuberculosis* bacteria and extend the time for negative BTA conversion. As many as 50% of patients with TB and diabetes remained positive on evaluation culture results in months 2-3 compared to those without DM. Some studies have also shown that diabetes mellitus may worsen TB treatment outcomes, specifically increasing the risk of death during TB treatment by as many as two times compared to patients without DM. Cases of TB with DM are also associated with an increased risk of cardiovascular complications and higher mortality in the first few months of TB treatment [8]. Some studies showed that the impact of diabetes on TB was that TB therapy tended to fail, and sufferers were less likely to die during therapy than non-diabetics [9].

Given the increasing worldwide DM epidemic, it is necessary to add DM prevention and control strategies to TB control programs and vice versa and to evaluate their effectiveness. The similarity of the two diseases can carry a risk of global spread, with severe implications for TB control.

## Conclusion

Screening for TB patients to find out comorbid DM is highly recommended for the eradication of TB treatment and sputum conversion and also to ensure diabetes control.
